# Promoting a Positive Relationship Between Physicians and Patients with Lyme Disease During Pregnancy and Parenthood

**DOI:** 10.3390/pathogens15040419

**Published:** 2026-04-13

**Authors:** Emma T. Hickman, Meagan E. Williams, Roberta L. DeBiasi, Sarah B. Mulkey

**Affiliations:** 1 Zickler Family Prenatal Pediatrics Institute, Children’s National Hospital, Washington, DC 20010, USA; 2 Division of Pediatric Infectious Diseases, Children’s National Hospital, Washington, DC 20010, USA; 3 Department of Pediatrics, The George Washington University School of Medicine and Health Sciences, Washington, DC 20037, USA; 4 Department of Neurology, The George Washington University School of Medicine and Health Sciences, Washington, DC 20037, USA

**Keywords:** tick-borne disease, Lyme disease, post-treatment Lyme disease, qualitative research, patient–physician relationship, in utero infection, Lyme infection-associated chronic illness

## Abstract

Patients with Lyme disease often face uncertainty when navigating the healthcare system during pregnancy and when seeking healthcare for their children exposed to Lyme disease in utero. Little is known about these families’ experiences. This qualitative study explored the perspectives of 27 parents in the United States who had acute or chronic Lyme-related diagnoses while pregnant. Semi-structured interviews were coded and thematically analyzed. Six themes characterized positive patient–physician interactions: (1) validation of patient experiences, (2) transparency, (3) willingness to learn, (4) shared decision making, (5) empathy, and (6) continuity of care. These findings offer guidance for clinicians counseling patients facing prognostic uncertainty related to Lyme disease and similarly complex conditions.

## 1. Introduction

Lyme disease (LD) is the most common tick-borne disease in the United States, primarily caused by the spirochete bacterium *Borrelia burgdorferi* in the Northeast United States or less commonly by *Borrelia mayonii* in the upper Midwest of the United States. Symptoms of Lyme disease can include a characteristic rash known as Erythema migrans, fever, arthralgias, and malaise early in the course with late manifestations of meningitis, carditis, and/or arthritis [1,2]. Post-Treatment Lyme Disease Syndrome (PTLDS) is less well-defined but the proposed definition set forth by the Infectious Diseases Society of America (IDSA) in 2006 includes the following components: (1) a diagnosis of Lyme disease fulfilling the case definition of the Centers for Disease Control and Prevention (CDC) with resolution or stabilization of the objective manifestations of LD, (2) onset of fatigue, musculoskeletal pain, or cognitive difficulties within 6 months of the diagnosis of Lyme disease, and (3) persistence of symptoms for at least a 6-month period after completion of antibiotic therapy [3]. This definition was reaffirmed in 2010 and has not been revised in further iterations of IDSA guidelines on LD [4,5]. Current evidence suggests that PTLDS is due to an ongoing autoimmune response after primary infection; however, like other infection-associated chronic illnesses (IACIs), the pathophysiology behind PTLDS is not yet fully understood [6]. The International Lyme and Associated Diseases Society (ILADS) has also identified “chronic Lyme disease” as a broader term which is distinguished from PTLDS by a greater number of inclusionary symptoms across multiple body systems and the suggestion of an ongoing *Borrelia* infection as the origin of persistent sequelae [7]. The National Academy of Science has set forth the term “Lyme Infection-Associated Chronic Illness” to encompass both PTLDS and chronic Lyme in an effort to fuel further investigation into these conditions and the similarities they may share with other IACIs such as long COVID [8]. In the face of ongoing controversy and uncertainty related to the origin, diagnosis, and treatment of lasting symptoms after Lyme disease, patients dealing with ongoing Lyme-associated symptoms face unique barriers as they navigate the health system [9,10,11].

In the last few decades, the number of Lyme disease cases has increased dramatically in the United States with some sources citing 476,000 new cases of LD per year and estimates of 10–20% of patients with LD developing PTLDS [12,13]. In light of these data, it is vital to understand the acute and chronic health risks associated with these conditions and their impacts on population health, including the effect on pregnant individuals and their children [14]. Cases of LD in pregnancy are rare, accounting for approximately 0.06% of cases reported to the CDC from 1992 to 2019, with ninety-one percent of them originating from fifteen high-incidence states and the District of Columbia [15]. Importantly, there is concern for underdiagnosis of LD in pregnancy, specifically in groups with low income and low educational attainment [16].

Currently, there is little data regarding the health outcomes of children exposed to Lyme disease in utero as well as the experiences of patients with Lyme-related diagnoses during pregnancy [14,16]. There is a general consensus that vertical transmission of Lyme disease is possible as in other spirochete infections such as syphilis and leptospirosis [14,15,16,17,18,19,20,21]. In pregnancies affected by Lyme disease, there are reports of spirochetes identified on placental pathology as well as in blood and tissue of stillborn fetuses and neonates who passed away shortly after birth [17,18,19]. Despite these findings, it remains unclear whether the presence of spirochetes in these tissues results in adverse effects [19]. While case reports and epidemiological studies have shown associations between Lyme disease during pregnancy and complications such as higher rates of miscarriage, stillbirth, preterm birth, and neonatal manifestations of hyperbilirubinemia, respiratory distress, and congenital anomalies, outcomes are variable, seeming to support the conclusion that there is no single congenital syndrome [16,19,20]. There does appear to be a consistent finding that untreated Lyme disease during pregnancy results in a higher frequency of symptoms in children, with some studies reporting increased incidence of rashes, unexplained fever, hypotonia, and respiratory distress compared to patients whose mothers received treatment [16,19]. Vaccines for Lyme disease are under development but not currently available [22,23]. Even with the information that has been gleaned so far from systematic reviews and cross-sectional surveys regarding Lyme disease in pregnancy, there is still much to learn about the impacts of LD on the fetus and long-term outcomes. Even more is unknown about the potential impacts of PTLDS and chronic Lyme, if any, on the pregnant individual.

Physicians often experience uncertainty in counseling patients with Lyme-related diagnoses in general due to variations in diagnostic practice in different settings (e.g., urgent care vs. primary care), online misinformation, and limited educational resources for both providers and patients [24]. Physicians treating pregnant women with Lyme-related diagnoses and their antenatally exposed children therefore face unique challenges in prognostic uncertainty compounded by potential frustration and discomfort. This study aimed to understand common experiences among patients with Lyme-related diagnoses while pregnant and parenting, with the goal of helping fetal and neonatal physicians and other maternal and pediatric healthcare providers promote a positive patient–physician relationship when interacting with families dealing with prognostic uncertainty surrounding Lyme disease and other complex conditions.

## 2. Materials and Methods

### 2.1. Recruitment

Participants were recruited through partnership with regional providers, Lyme disease advocates, and online interest groups. Participants were eligible for this study if they met the following criteria: (1) older than 18 years of age, (2) living in the United States, and (3) reported being diagnosed by a healthcare provider with LD, PTLDS, and/or chronic Lyme while they were pregnant, or if they had a diagnosis of PTLDS/chronic Lyme before becoming pregnant with ongoing symptoms during pregnancy. All participants completed a screening call to confirm eligibility.

### 2.2. Qualitative Interview

Participants completed a semi-structured qualitative interview during which they were asked about their experiences navigating systems of care for themselves and their children in the context of their Lyme-related condition. Interviews were recorded, transcribed, and manually coded via inductive content afnalysis by two team members independently utilizing the coding software Dedoose version 10.0.34 [25,26]. The codes were then reviewed jointly and agreed upon by the group. Codes relating to the patient–physician relationship were examined and consolidated, with a focus on comparing and contrasting the most frequent sentiments expressed by the participants. The codes were re-examined and redefined as needed for accuracy, as each interview was coded and memos were utilized to link overarching themes as they emerged using an inductive thematic analysis framework [27].

### 2.3. Quantitative Survey

Participants additionally completed a one-time quantitative REDCap survey including demographic and socioeconomic information, parent medical history, pregnancy history, and child medical history. Study data were collected and managed using REDCap electronic data capture tools hosted at Children’s National [28]. Descriptive statistics for the patient cohort were generated based on survey data collected.

## 3. Results

Twenty-seven participants were enrolled and completed qualitative interviews. Patient characteristics and additional information regarding Lyme diagnoses and pregnancy/parenting experience are discussed below and summarized in Table 1 and Table 2. Six themes emerged as vital components of a positive patient–physician interaction in the context of seeking treatment for a Lyme-related diagnosis during pregnancy and beyond: (1) validation of patient experiences, (2) transparency, (3) willingness to learn, (4) shared decision making, (5) empathy, and (6) continuity of care.

### 3.1. Patient Characteristics

The mean age of enrollment was 38.5 years (range: 23.2 to 65.4 years) with a few participants reflecting on pregnancy experiences decades prior. Participants largely identified as white, and two identified as multiracial. One participant identified as non-binary. The majority of participants had completed a bachelor’s degree or higher level of education and resided in the northeast region of the United States. Most participants had a diagnosis of PTLDS during pregnancy; forty-eight percent of participants received their diagnosis > 10 years prior to enrollment. Most participants had at least two or more children and at least one child 5 years of age or younger. Additional participant characteristics are described in Table 1 with details about Lyme diagnosis and pregnancy/parenting experience in Table 2.

### 3.2. Six Themes of a Positive Patient–Physician Interaction

#### 3.2.1. Validation of Patient Experiences

One hundred percent of participants reported being dismissed by healthcare providers with regards to their symptoms, diagnoses, and/or experiences in the healthcare system. In a variety of settings, from primary care offices to emergency room visits to specialty care visits, participants felt their experiences were minimized, attributed to stress, or outright denied. These experiences led to distrust of the medical system and providers, highlighting the stark contrast between physicians who would take the time to listen to them and validate their experiences versus those that did not.

*“What I liked most out of anything, like actually she could be awful and she could do a hack job, but our first appointment this time around she…was like, ‘[Participant Name], could I just ask, like, what do you think is going on?’ And like, it meant the world to me. [voice breaks] ‘Cause like, nobody had asked me that in so long, or maybe ever…just to feel like I am the expert in my body”*

*“[Provider] listens. And she validates you, so you don’t think that you’re crazy.”*

*“I think if [providers] just believed [patients], that would be gigantic. Because so many people don’t even believe it. But then when you hear somebody that says, ‘oh, my niece has Lyme, I totally understand’ or… ‘oh, Lyme’s just got to be hard, right? I’ve seen a lot of patients with that.’ It’s like, that just verifies that you’re not crazy… And so just to be believed, especially as a pregnant mom, that’s huge.”*

*“If somebody just believes you, you feel better about talking to them with it too.”*

Participants expressed gratitude towards providers who believed what they had been through. Participants went to great lengths to continue receiving their care from those providers as well as suggesting them to friends or family members dealing with similar situations.

#### 3.2.2. Transparency

Many participants valued their provider being transparent regarding their knowledge and experience with Lyme-related diagnoses during pregnancy (or lack thereof) rather than claiming to know all the answers. Openness from providers was mentioned by seventy percent of participants as an important characteristic in patient–provider interactions. Several participants experienced provider dismissal regarding their concerns about the effects of LD and PTLDS on pregnancy without clear evidence to support the provider’s assertions.

*“I would so much have rather heard ‘We’re not sure, but we’re going to send you to someone else’… I don’t care if you know everything, I just want you to be nice. And help, help if you can, if you can’t, just be kind… There’s never a reason to be mean to anyone. Humans are humans. Everyone deserves dignity, respect, courtesy, and like, I would think that the doctor-patient relationship, you would find an increased interest in providing that. But I have not.”*

#### 3.2.3. Willingness to Learn

Participants commonly recounted visits to providers who had never taken care of a patient with Lyme-related diagnoses during pregnancy or who assumed there was no increased risk of complications because they had never heard about them. Providers who were willing to not only admit inexperience but also to commit to familiarizing themselves with the available literature were extremely valued by participants who had often experienced quick dismissal by others in the past.

*“As a practitioner, we need to be open minded and realize that again, we don’t have all the answers. And it’s OK to learn new things and to change your mind… everyone needs to be open minded, and I think just realize that like overall research of most, you know, bacteria is vastly unknown. We love thinking that we have all the answers, but we don’t.”*

All participants pursued additional sources of information regarding their Lyme-related diagnosis beyond what their doctor had shared. A number of participants took it upon themselves to search the medical literature relating to Lyme disease during pregnancy when they felt their providers were unwilling to do so.

*“But we are so new at this, even only a few decades into really being able to put things under the microscope, we still don’t have many of the answers that we need. And you know, so just as much as it’s on the patient to realize that, I think it also comes down to doctors needing to rethink their approach to it… am I getting the most up-to-date information? Am I doing my due diligence when I took this role to make people healthy? Is that ultimately what I’m doing, or am I putting my own feelings about it onto my client? And so I encourage everyone to you know, think deeper and look at what’s out there.”*

*“We need more people with a broader perspective that are willing to relearn what they’ve been taught. That are open to saying ‘I’m wrong, maybe we were wrong and maybe we need to revisit this.’ … if you’re going to be a good doctor, I mean, especially with all the people that are getting [Lyme] in my area… they might want to educate themselves a little bit more because this is not going away.”*

#### 3.2.4. Shared Decision Making

When it came to testing, treatment, and additional management considerations, participants highly valued being included in the decision making given the lack of evidence-based guidelines for management of patients with Lyme-related diagnoses during pregnancy. Collaboration with providers was mentioned in virtually all interviews, with participants recounting either positive experiences or wishing that their doctors had been more amenable to these approaches. Providers who talked about risks and benefits of treatments and proposed various approaches to symptom management were more likely to make a positive impression on participants.

*“…being allowed to make decisions myself. Being presented with options and allowed to make decisions for what’s best for me instead of just [the provider] being like, ‘well this is what I’m going to offer you.’”*

In situations where participants did not encounter this collaboration from providers, they were often left feeling alone in their management and were more likely to take it upon themselves to research treatments and ways to manage their symptoms.

*“It is great to take control of your own health. However, it would be even better if you had someone that was that expert right in front of you and able to work through it together, not like expecting someone to fix you but working through it together instead of relying on books of, you know, all the research that’s out there. I think it would have been really helpful to have someone that has experience of finding that custom plan for someone experiencing Lyme.”*

#### 3.2.5. Empathy

Participants described a high symptom burden relating to their Lyme diagnoses, resulting in high emotional distress and significant limitations in functioning. In the process of seeking care, many participants encountered providers who made them feel like their symptoms were “all in their head” and failed to recognize or attempt to understand what their patients were going through. Over two-thirds of participants reported feeling “not believed” by providers and one hundred percent had an experience where they felt “dismissed” by a provider. This was significantly damaging to the patient–physician relationship, even if the provider was someone with whom the patient was well established.

*“I just went to my doctor recently and he’s like to me, ‘What are you doing? You’re turning yourself upside down for no reason. Lyme is not a big deal, it doesn’t kill you.’ He’s like ‘I’ve had-I have patients coming in with, you know, end stage, whatever cancers. And this is not a big deal. Go live your life. Forget about that you have Lyme. Go have kids and don’t think about it.’ And I’m like, no, no, no. You don’t understand. Like, I’m not looking for more problems in my life. I don’t want to be fatigued, like I literally want to be a great mother to my kids, and I don’t want to be always laying down. I want to absorb the food I eat, like, I’m not making this stuff up… He thought it was all in my head. He’s like, ‘It’s all in your head. Just go and get up.’”*

Even in the absence of provider expertise, empathy and recognition of patient hardships went a long way to establish goodwill with participants.

#### 3.2.6. Continuity of Care

The diagnostic journey was something that participants reflected on frequently, with many detailing how hard it was for their symptoms to be recognized as reflective of Lyme disease, especially in the absence of a known tick bite. The participants expressed feeling burdened to explain their diagnosis and concerns each time they went to a new provider, particularly during pregnancy, which made it difficult to establish a trusting rapport. While approximately three-quarters of participants experienced improvement of their Lyme-related symptoms during pregnancy, the most common co-occurring codes with pregnancy were “unknown”, “fear”, and “anxiety” and one hundred percent of participants expressed concerns about congenital transmission of Lyme disease. When they brought up concerns about Lyme, they would get different responses from different providers, many of whom would not feel Lyme was an important part of their medical history and pregnancy.

*“So you would see a different doctor almost every time you went. And I almost brought it up every [prenatal] appointment because I knew I wasn’t feeling well, and then it would just be like, ‘well, you’re older,’ you know, I would always get those reasons. And then if I went back—and I always mentioned Lyme, like most I would say 90% of the time—it wasn’t even written down. So they didn’t see it as even a reason to go deeper to why I wasn’t feeling well.”*

*“Honestly, so the person that I had gone to to help me get the diagnosis ended up not really sticking around to stay, you know, for the treatment of it… We paid all this money for someone who’s private to be like, ‘well I don’t know what to do for you’ even though she, you know, kind of prompted us and to be like ‘hey I can help you out’ and then was like, ‘ohh actually I can’t help you.’”*

## 4. Discussion

Providers who practiced validation of patient experiences, transparency, willingness to learn, shared decision making, empathy, and continuity of care were more likely to leave a positive impression on patients with Lyme-related diagnoses during pregnancy. While these are important practices in all patient–physician relationships, they are particularly important to keep in mind in situations where there are high levels of uncertainty regarding future outcomes. Pregnancy is naturally a time when parents are worried about the future, and complications can cause significant stress and anxiety. It is important for providers to recognize that data are limited regarding Lyme-related diagnoses in pregnancy and the potential impacts on maternal health, pregnancy complications, and child health and development. While the frequency and severity of LD-related complications during pregnancy have yet to be consistently quantified, this does not equate to absence of risk [19]. Validation of patient experiences is an especially salient quality in the patient–physician relationship in this context as all participants in our study shared feeling dismissed by healthcare providers with regards to their symptoms, concerns, and lived experiences. Expertise in this area is not yet achievable given the uncertainties that remain, highlighting the need for transparency by healthcare providers in counseling and management. Willingness to learn is a quality that is also supported by patient-identified priorities reported by Omar et al., including better-informed healthcare providers and more public awareness [18].

Our study has some limitations that highlight important considerations for future studies. The recruitment method for this study presents the potential for self-selection bias. While this sample size is typical for qualitative research, the small sample size and lack of diversity in racial, ethnic, and educational backgrounds of participants also limit our ability to draw conclusions that can be applied across sociodemographic groups. Race, ethnicity, and other demographic characteristics of both patient and provider can influence the communication and relationship between them [29,30,31], and research suggests that there are differences in the prevalence, knowledge, and manifestations of Lyme disease across racial and ethnic groups [32,33]. To fully inform provider training and patient advocacy initiatives based on this work, future research should focus on understanding the experiences of non-white patients navigating the patient–provider relationship at this intersection of Lyme disease and pregnancy. Additionally, several participants recounted experiences from pregnancies years earlier, which allows for potential recall bias. However, the fact that these interactions with clinicians have remained ingrained in participants’ memories for over 10 years can also be interpreted as evidence of the importance of patient-centered, compassionate care in all interactions with patients.

Despite these limitations, it is clear that participants placed high value on the aforementioned six practices in helping them feel seen and cared for. Our findings can provide important guidance for maternal, fetal, and neonatal physicians in counseling and treating patients with Lyme-related diagnoses during pregnancy and their families, in addition to informing the approaches of physicians in other lesser understood conditions such as other infection-associated chronic illnesses affecting the parent–baby dyad.

## Figures and Tables

**Table 1:** Participant characteristics (n = 27).

**Age (years)**	
Mean (SD) age at enrollment [range]	38.5 (9.0) [23.2–65.4]
**Race/Ethnicity**	
Single race, white	25 (93%)
Multiracial	2 (7%)
Non-Hispanic	27 (100%)
**Gender Identity**	
Woman	26 (96%)
Non-binary	1 (4%)
**Highest Education Completed**	
High school	1 (4%)
Community/technical college	9 (33%)
Bachelor’s degree	10 (37%)
Master’s degree	5 (18%)
Doctoral degree	1 (4%)
Other/unknown	1 (4%)
**Region of Residence (United States)**	
Northeast	12 (44%)
Midwest	4 (15%)
South	7 (26%)
West	4 (15%)

**Table 2:** Lyme and pregnancy/parenting experience.

**Lyme-related diagnosis**	
Primary diagnosis during pregnancy	
Acute Lyme disease	6 (22%)
PTLDS or chronic Lyme	21 (78%)
Timing of first Lyme-related diagnosis	
<5 years ago	7 (26%)
5–10 years ago	7 (26%)
>10 years ago	13 (48%)
**Pregnancy/parenting experience**	
Number of living children at the time of study	
1	8 (30%)
2	11 (41%)
3	5 (19%)
4 or more	3 (11%)
Age of children	
At least one child 5 years or younger	20 (74%)
All children > 5 years old	7 (26%)

## Data Availability

Data can be requested by sending correspondence to the corresponding author.

## Abbreviations

The following abbreviations are used in this manuscript:

LD	Lyme Disease
PTLDS	Post-Treatment Lyme Disease Syndrome
IDSA	Infectious Diseases Society of America
CDC	Centers for Disease Control and Prevention
ILADS	International Lyme and Associated Diseases Society
IACI	Infection-Associated Chronic Illness
